# Functional Identification and Genetic Analysis of O-Antigen Gene Clusters of Food-Borne Pathogen *Yersinia enterocolitica* O:10 and Other Uncommon Serotypes, Further Revealing Their Virulence Profiles

**DOI:** 10.4014/jmb.2402.02044

**Published:** 2024-07-15

**Authors:** Bin Hu, Jing Wang, Linxing Li, Qin Wang, Jingliang Qin, Yingxin Chi, Junxiang Yan, Wenkui Sun, Boyang Cao, Xi Guo

**Affiliations:** 1 Shandong Center for Disease Control and Prevention, 16992 City Ten Road, Jinan 250014, Shandong, P.R. China; 2 TEDA Institute of Biological Sciences and Biotechnology, Nankai University, 23 Hongda Street, TEDA, Tianjin 300457, P.R. China; 3 Disease Prevention and Control Center of Ganzhou District, 27 Xianfu Street, Ganzhou District, Zhangye City, Gansu Province, P.R. China

**Keywords:** *Yersinia enterocolitica*, serotype, O-antigen gene cluster, virulence gene, pathogenicity

## Abstract

*Yersinia enterocolitica* is a globally distributed food-borne gastrointestinal pathogen. The O-antigen variation-determined serotype is an important characteristic of *Y. enterocolitica*, allowing intraspecies classification for diagnosis and epidemiology purposes. Among the 11 serotypes associated with human yersiniosis, O:3, O:5,27, O:8, and O:9 are the most prevalent, and their O-antigen gene clusters have been well defined. In addition to the O-antigen, several virulence factors are involved in infection and pathogenesis of *Y. enterocolitica* strains, and these are closely related to their biotypes, reflecting pathogenic properties. In this study, we identified the O-AGC of a *Y. enterocolitica* strain WL-21 of serotype O:10, and confirmed its functionality in O-antigen synthesis. Furthermore, we analyzed *in silico* the putative O-AGCs of uncommon serotypes, and found that the O-AGCs of *Y. enterocolitica* were divided into two genetic patterns: (1) O-AGC within the *hemH-gsk* locus, possibly synthesizing the O-antigen via the Wzx/Wzy dependent pathway, and (2) O-AGC within the *dcuC-galU-galF* locus, very likely assembling the O-antigen via the ABC transporter dependent pathway. By screening the virulence genes against genomes from GenBank, we discovered that strains representing different serotypes were grouped according to different virulence gene profiles, indicating strong links between serotypes and virulence markers and implying an interaction between them and the synergistic effect in pathogenicity. Our study provides a framework for further research on the origin and evolution of O-AGCs from *Y. enterocolitica*, as well as on differences in virulent mechanisms among distinct serotypes.

## Introduction

*Yersinia enterocolitica* is a Gram-negative, food-borne, zoonotic pathogen with worldwide distribution [1]. It is the causative agent of yersiniosis, which is mainly characterized by gastrointestinal disorders; however, extraintestinal systemic manifestations and septic complications have also been recognized occasionally [2]. According to their biochemical features and pathogenic properties, *Y. enterocolitica* strains are divided into six biotypes: the highly pathogenic biotype 1B; the weakly pathogenic biotypes 2–5; and the non-pathogenic biotype 1A [3]. In addition, with regard to the antigenic scheme, more than 70 serotypes of *Y. enterocolitica* have been identified to date [4]. Among these, only 11 serotypes have been reported to be associated with human yersiniosis, with O:3, O:9, O:8 and O:5,27 being the most prevalent, according to clinical evidence [4, 5].

In Gram-negative bacteria, lipopolysaccharide (LPS) is a major component of the outer membrane. The full LPS molecule includes three structurally distinct regions: the lipid A; an oligosaccharide core; and the O-antigenic polysaccharide (O-antigen) [6]. The O-antigen is made of oligosaccharide repeats (O-units) each consisting of two to eight different monosaccharide residues (heteroglycans) or, in some bacteria, identical sugars (homoglycans)[7]. The O-antigen is the most variable LPS component in terms of composition and structure, and thus provides the molecular basis for serotyping, one of the main useful tools for epidemiological, diagnostic, and phylogenetic purposes [8]. Enzymes involved in O-antigen synthesis are encoded in a number of genes, which are always located within a chromosome-encoded locus, namely, the O-antigen gene cluster (O-AGC), and the high variability of O-antigens results normally from the genetic diversity of O-AGC [9,10]. The synthesis of O-antigen depends on one of two classical pathways: the Wzx/Wzy dependent pathway [11] and the ABC transporter (Wzm/Wzt) dependent pathway [12].

The O-antigen chemical structures have been reported for several *Y. enterocolitica* serotypes, including O:1 to O:3, O:5,27, O:6, and O:8 to O:10 [13-18], and the O-AGCs are known for serotypes O:3, O:9, O:8, O:5 and O:5,27 [19]. In serotype O:8, the O-AGC is located between the *hemH* and *gsk* genes. In other serotypes, however, the *hemH-gsk* locus is occupied by the outer core gene cluster, and the O-AGCs are located elsewhere, always along with several transposase genes; this probably indicates a hotspot region of lateral gene transfer [20, 21]. Since serotyping is one of the main characteristics of *Y. enterocolitica*, a number of PCR-based methods have been developed for the detection of clinically prevalent serotypes of *Y. enterocolitica* with higher efficiency and specificity based on the comprehensive elucidation of their O-AGCs [19, 22, 23]. In addition to the main serotypes which are strongly associated with human infection, a few minor serotypes have also been reported as causal agents in sporadic cases [24, 25].

Besides the O-antigen that has been demonstrated to be a virulence factor in *Y. enterocolitica* [26, 27], pathogenic *Y. enterocolitica* strains harbor many other chromosome- and plasmid-encoded virulence determinants that are essential for pathogenesis [28]. The main chromosomal virulence genes include the following: *ail*, encoding the protein which plays a key role during attachment and invasion processes, and which also confers serum resistance [29]; *inv*, whose product is required in the early phase of infection [30]; *myfA*, encoding a fibrillar submit of Myf, an important factor at the beginning of infection [31]; and *yst*, encoding an enterotoxin [32]. In addition, several genes located within a virulence plasmid, pYV, are directly involved in the pathogenicity of *Y. enterocolitica*, including *yadA*, encoding *Yersinia* adhesin, and the *yop* virulon genes, encoding a type III secretion system (T3SS), namely, Ysc-Yops, translocator YopB/D, control element YopN, and effector YopE/H/M/O/P/T [33]. Normally, high-virulence strains of biotypes 1B and low-virulence strains of biotypes 2–5 carry the chromosome-encoded virulence markers *ail*, *inv*, and *ystA*, in addition to pYV, for their full virulence expression. In contrast, nonvirulent strains belonging to biotype 1A lack pYV-encoding virulence factors, but mainly possess *ystB*, another type of *yst* gene [19]. Many studies showed that *Y. enterocolitica* strains of different biotypes exhibit disparate pathogenic properties and virulence profiles [34-36]. However, the link between serotypes and virulence gene distribution in *Y. enterocolitica* has not yet been reported.

During the routine epidemiological surveillance, a *Y. enterocolitica* strain, which was numbered WL-21, was isolated from the stool sample of a chicken by the Shandong Centre for Disease Control and Prevention. Further agglutination test by using antisera (provided by Chinese Center for Disease Control and Prevention) showed that WL-21 was serotype O:10, and biotyping test according to a previous study [37] showed that this isolate belonged to biotype 1A. As an uncommon serotype, neither the O-AGC nor the virulence patterns of O:10 was revealed. The first objective of the present study was to characterize the O-AGC of WL-21, and certify its role in O-antigen synthesis. Secondly, through comprehensive *in silico* analysis of the O-AGCs, we produced a blueprint that may contribute to elucidating the origin and evolution of O-AGC in *Y. enterocolitica*. Finally, we sought to recognize any interactions between O-antigen and other virulence factors in the pathogenicity of *Y. enterocolitica*.

## Materials and Methods

### Bacterial Strains, Plasmids, and Growth Conditions

Details of WL-21 and its derivatives, plasmids, and primers which were used in this study are given in Table 1. All strains used for sequencing and gene manipulation were cultured in Luria–Bertani (LB) medium at 30°C. When necessary, the cultures were supplemented with chloramphenicol (25 μg/ml).

LB medium (Cat. no. R20214) and chloramphenicol (Cat. no. S17022) were purchased from YuanYe Bio-Technology Co., Ltd. (China). The DNA extraction kit (Cat. no. DP302-02) was purchased from Tiangen Biotech Co., Ltd. (China). High-Fidelity DNA Polymerase (Cat. no. M0530S), restriction enzymes (Cat. nos. R3142V and R3104V) and T4 DNA ligase (Cat. no. M0202V) were purchased from New England Biolab (USA). Sucrose (Cat. no. A610498), L-arabinose (Cat. no. A610071), and reagents for LPS preparation and sodium dodecyl sulphate–polyacrylamide gel electrophoresis (SDS-PAGE) were purchased from Sangon Co., Ltd. (China). Primers were synthesized by Genewiz (China).

### Genome Sequencing and Annotation

The genomic DNA of *Y. enterocolitica* strain WL-21 was extracted from 5 ml of the overnight bacterial culture. Then, the genomic DNA was sheared, polished, and prepared using the Nextera XT DNA library prep kit (Illumina, USA). Genomic libraries containing 500-bp paired-end inserts were constructed, and sequencing was then performed using Solexa sequencing technologies (Illumina) to produce approximately 100-fold coverage. The obtained reads were assembled using the *de novo* genome assembly program Velvet to generate a multi-contig draft genome [38]. For gene prediction and annotation, Prokka (v1.12) was used [39], and for additional annotation, the assembled sequences were searched against GenBank non-redundant (NR), UniProt, and Pfam database using BLAST (v2.5.0+) [40]. The TMHMM v2.0 analysis program (http://www.cbs.dtu.dk/services/TMHMM-2.0/) was used to identify potential transmembrane domains within the protein sequences. The sequence data were submitted to GenBank under accession number PP132002. Meanwhile, the genome sequence of a strain IP2222 (O:36) was downloaded from GenBank (GCA_000285015.1) and annotated using the above procedures.

### Construction of Plasmids and Strains

Gene deletion in the WL-21 chromosome was performed according to a two-step homologous recombination with pRE112 containing the *sacB* counter-selectable marker, as described previously [41]. The upstream and downstream fragments of the *wzm* gene were amplified from the WL-21 genome using primer pairs upF/upR and downF/downR. Next, the two fragments were combined with each other using primers upF and downR, followed by fusion with the linearized pRE112 to yield the suicide plasmid pRE112-updown, which was introduced into the S-17 strain by electroporation, generating the donor strain S-17-pRE112-updown. For conjugation, S-17-pRE112-updown and the recipient strain WL-21 were grown at 30°C with shaking overnight. Then, the cultures were diluted as a ratio of 1:100 into fresh LB medium and incubated at 30°C with shaking until the optical density at 600 nm (OD_600_) reached approximately 0.6. Next, the donor and the recipient strains were mixed at a ratio of 3:1 (v/v), and the mixture was resuspended in 100 μl of LB medium and was spotted on a LB plate with incubation at 30°C for 48h. After conjugation, the cells were collected and transferred to chloramphenicol containing LB plate to screen for clones via a single crossover event. The growing clones were then transferred into fresh LB medium and incubated at 30°C overnight to induce the second homologous recombination. The overnight culture was diluted and spread on LB plates containing 10% sucrose and grown at 30°C for 24 h. Finally, the resultant clones were transferred onto LB plates and LB plates with chloramphenicol simultaneously, and clones sensitive to chloramphenicol were selected and confirmed by PCR and sequencing.

For the complementation test, the *wzm* gene was amplified from the WL-21 genome using primers wzm-cF and wzm-cR. The PCR fragment was digested with restriction enzymes KpnI and HindIII, and the digested fragment was cloned into pBAD33, which was also treated with the same enzymes, resulting in plasmid pBAD33-wzm. Expression of the cloned *wzm* gene is under the control of the P_BAD_ promoter, which could be activated by arabinose. Then, pBAD33-wzm was introduced into WL-21Δ*wzm* by electroporation, generating the complementary strain WL-21Δ*wzm*::*wzm*.

### LPS Extraction and SDS-PAGE Analysis

Strains were grown overnight at 22°C with shaking, and cultures were diluted into 20 ml of fresh LB broth at a ratio of 1:100 and incubated at 22°C to mid-log phase at a final OD600 = 0.8. To induce *wzm* expression under the control of pBAD33, L-arabinose (0.5 mg/ml) was added to cultures at the OD_600_ = 0.4, and the cultures were incubated continuously to mid-log phase at the OD_600_ = 0.8. Then, LPS used for SDS-PAGE analysis was prepared using the hot aqueous-phenol method, as previously described [42]. The extracted LPSs were separated using 12%SDS-PAGE at 50 V for 30 min and 100 V for 2 h; subsequently, they were visualized by silver staining using the Fast Silver Stain Kit (Cat. no. P0017S; Beyotime, China), according to the manufacturer’s protocol. The gel image was captured using a GS900 calibrated densitometer (BioRad Laboratories, USA).

### Analysis of Putative O-AGCs and Virulence Marker Profiles

Genomes of those isolates with the serotypes assigned to by the other submitters were downloaded from the GenBank database. Putative O-AGCs of serotypes were characterized using the in-house Bacterial Surface Polysaccharide Gene Database. In general, query genome sequences in GenBank format were searched against the database using BLASTp with the cutoff %coverage > 60 and %identity > 30. An O-AGC candidate could be defined using the following criteria: (1) the smallest number of successive genes is six; (2) the number of successive genes annotated “No hits” is no more than three; and (3) there must be glycosyl transferase gene(s), in addition to *wzm*/*wzt* or at least one of *wzx* and *wzy* genes. Schematic diagram of genes (Fig. 3) was generated using ChiPlot [43].

The downloaded genome sequences, along with that of WL-21, were also compared with the selected virulence genes, including *ail* [29], *inv* [30], *myfA* [31], *ystA/B* [32], *yadA* and *yop* virulon [33], *ymoA* [44], *yplA* [45], *fliA* and *flh/C/D* [46], *fyuA* and *ybtP/Q* [47], and *ysa* genes [48], which have been identified previously. Nucleotide sequences were compared using BLASTn, and genes with >90% coverage match and >85% identity match were classified as present. Isolates were clustered according to their virulence profiles, and the pattern was visualized by ChiPlot [43].

## Results and Discussion

### The Putative O-AGC of WL-21 Correlates Well with the O-Antigen Structure of *Y. enterocolitica* O:10

**Fig. 1:**
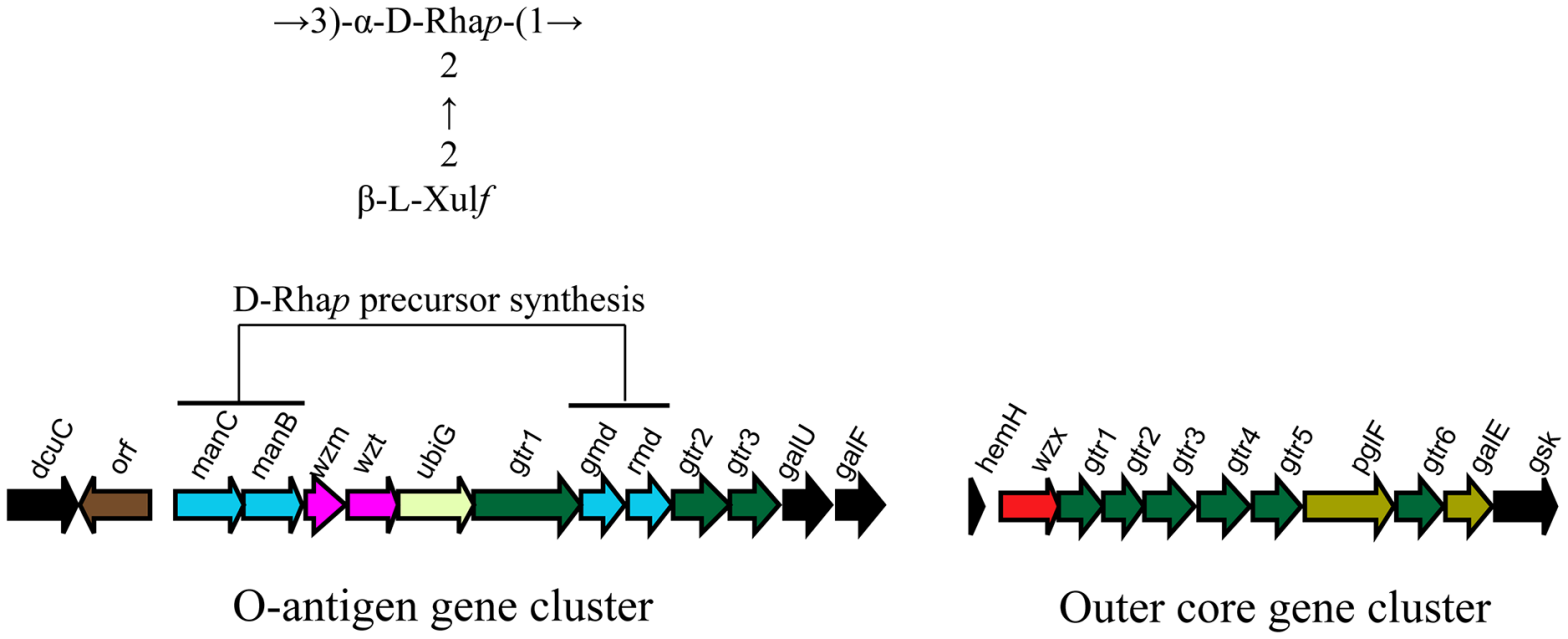
Representation of the O-antigen genetic cluster of *Y. enterocolitica* WL-21, with the O-antigen structure of serotype O:10 [18] shown at the top (left), as well as the outer core gene cluster (right).

The putative O-AGC of WL-21 is 16,137 bp in length, and consists of 11 open reading frames (*orfs*). These *orfs* were located between the *dcuC* and *galU-galF* genes, and the transcribed direction of *orf2* to *11* was from *dcuC* to *galU*, with the exception of *orf1* just downstream of *dcuC* (Fig. 1), in line with the evidence for O:3 and very similar to that for O:9.

Details of proposed functions of the 11 *orfs* were summarized in Table 2. Orf1, a TerC family protein (Pfam03741), was assigned to the function of transporter in O:3 and O:8. TerC protein may be implicated in resistance to tellurium [49]; however, no involvement of this protein in O-antigen synthesis has yet been reported. Orf2, 3, 8, and 9 were annotated as mannose-1-phosphate guanylyltransferase (ManC), phosphoglucomutase (ManB), GDP-mannose 4,6-dehydratase (Gmd), and GDP-6-deoxy-D-talose 4-dehydrogenase (Rmd), respectively. These four enzymes, along with mannose-6-phosphate isomerase (ManA), whose coding gene is always outside the O-AGC, are responsible for the synthesis of GDP-D-rhamnose (GDP-D-Rha*p*), the nucleotide precursor of D-Rha*p* [50]. This is consistent with the existence of D-Rha*p*, which is the only sugar component in the backbone of O:10-antigen. Orf4 and 5 were proposed as the ABC transporter permease (Wzm) and the ABC transporter ATP-binding protein (Wzt), respectively, suggesting that it is very likely that the O-antigen of WL-21 was generated via the ABC transporter dependent pathway. *orf6*, just downstream of *wzt*, was annotated a methyltransferase gene. This is very common in O-AGCs containing *wzm/wzt* genes, and its function has been identified as adding a methyl group to the non-reducing terminus of the O-antigen, thus halting polymerization and regulating chain length [51, 52]. Finally, Orf7, 10, and 11 were all recognized as glycosyl transferases. These enzymes could presumably be involved in the sequential transfer of GDP-D-Rha*p* to D-Rha*p* polymers, forming the backbone, and the transfer of L-xylulose (L-Xul*f*) to D-Rha*p*, forming the side-chain linkage L-Xul*f*-(β2→2)-D-Rha*p*. However, the exact functions of these glycosyl transferases should be confirmed biochemically in the future.

We also observed the *hemH-gsk* locus at the WL-21 genome (Fig. 1), within which the genetic organization was identical to the outer core gene clusters of O:3 and O:9. This finding also indicated that the genetic elements between *dcuC* and *galU-galF* were the candidates for WL-21 O-antigen synthesis genes.

### Deletion and Complementation Test Confirmed the Functionality of WL-21 O-AGC

**Fig. 2:**
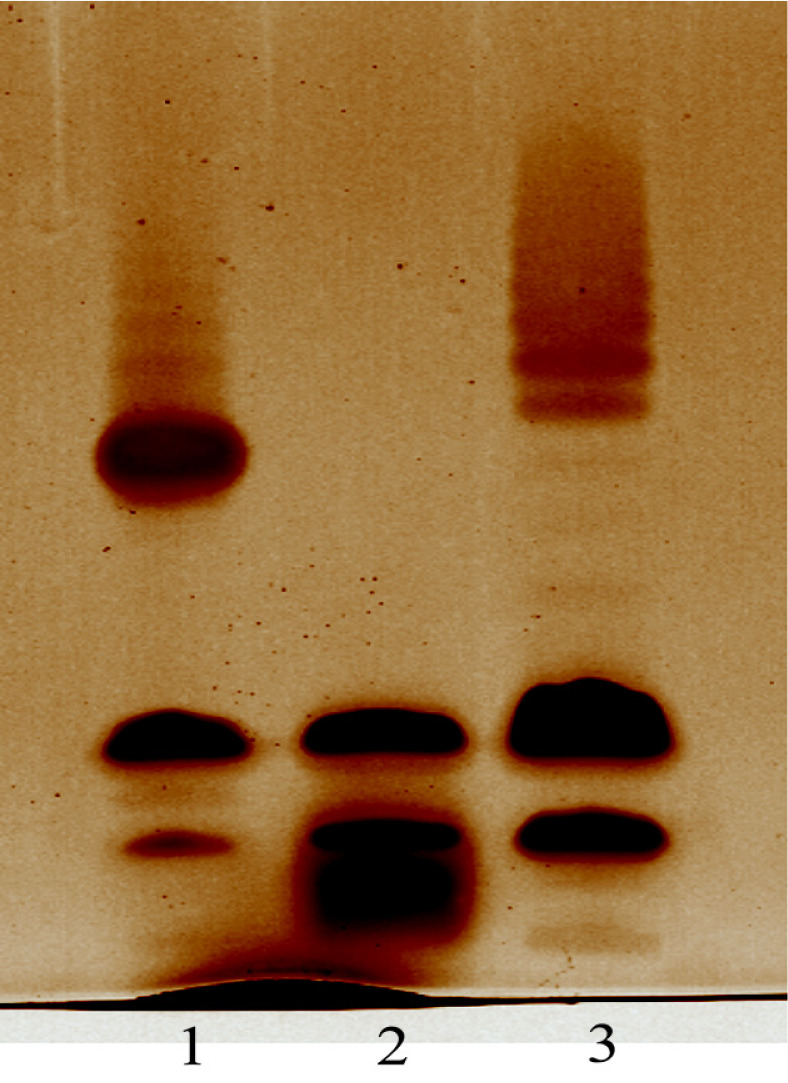
LPS profiles of WL21 and its derivatives. LPS extracts were electrophoresed on sodium dodecyl sulfate–polyacrylamide gel electrophoresis gels, and stained by silver staining. Lane 1, WL21 wide-type strain; lane 2, WL-21Δ*wzm*; and lane 3, WL-21Δ*wzm*::*wzm*.

To confirm the role of *dcuC-galU-galF* locus in O-antigen synthesis, a *wzm* knockout strain WL-21Δ*wzm* was constructed. As can obviously be seen in the LPS profile, the WL-21 wide-type strain exhibited a complete LPS, while WL-21Δ*wzm* only generated an O-antigen-depleted LPS (Fig. 2). Within the ABC transporter dependent pathway, the translocation of O-antigen is mediated by the integral membrane protein (Wzm)/hydrophilic protein complex (Wzt) [53]. Therefore, WL-21Δ*wzm* lost the ability to produce the Wzm/Wzt complex for O-antigen translocation, thus only the lipid A-core band was generated without O-antigen. Moreover, the complete LPS profile could be restored from the *wzm* complemented strain, WL-21Δ*wzm*::*wzm*. However, we noticed that the chain-length of O-anitgen increased slightly in WL-21Δ*wzm*::*wzm* compared to that in the wide type strain (Fig. 2). There is no clear-cut explanation, as polysaccharide structural studies always tend to focus on repeating units, rather than chain-length regulation. A study demonstrated that in *Escherichia coli* O9a, a prototype for the biosynthesis of O-antigens by the ABC transporter dependent pathway, the occurrence of distinct chain-length of O-antigens was dependent on the relative concentration of two enzymes, the glycosyl transferase WbdA and the bifunctional kinase–methyl transferase WbdD [54]. As a methyltransferase UbiG and three glycosyl transferases were also annotated in WL21, we propose that there might be similar chain-length regulating mechanism in *Y. enterocolitica*. Together, these results still proved that the *dcuC-galU-galF* locus is the O-AGC of WL21, and that the WL21 O-antigen is synthesized by the ABC transporter dependent pathway.

### The Putative O-AGCs of Most *Y. enterocolitica* Are Generally Divided into Two Genetic Patterns

A total of 137 *Y. enterocolitica* isolates from GenBank were assigned to certain serotypes by the submitters, the majority of which are prevalent serotypes with their O-AGCs being reported before this study: 41 for serotype O:3, eight for serotype O:5, 17 for serotype O:5,27, ten for serotype O:8, and 31 for serotype O:9. The remainder are isolates with uncharacterized O-AGCs, including isolates of serotypes O:1, O:2, O:4, O:6, O:7, O:13, O:19, O:21, and O:36. We next derived the putative O-AGCs from their genomes. Our analysis indicated that (1) isolates with the same serotype possess identical genetic organization within the putative O-AGC; and (2) most serotypes exhibit unique O-AGC profiles with the exception of O:1, O:2, O:7 and O:19.

**Fig. 3:**
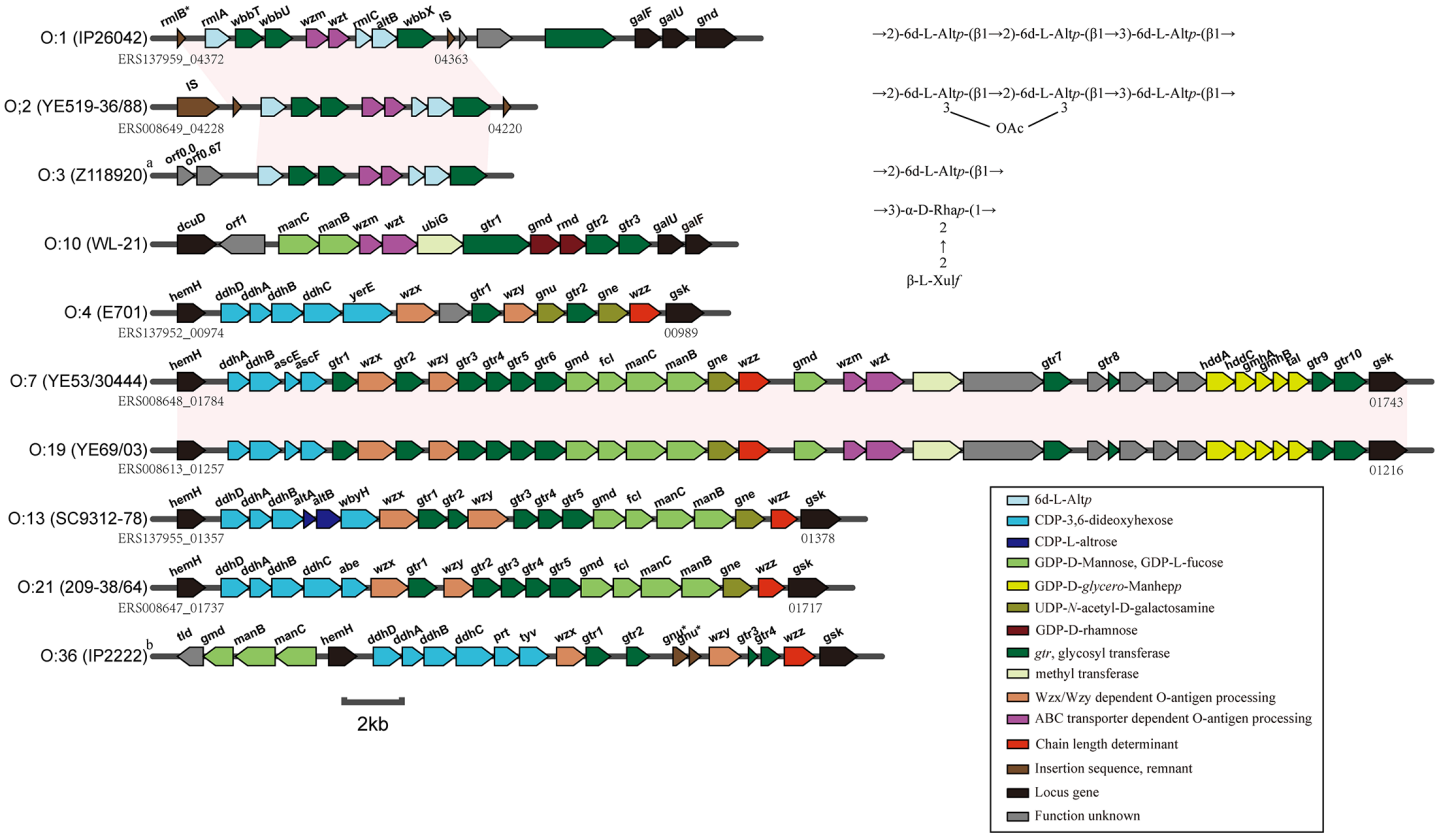
Representation of the O-antigen genetic clusters that have not been reported previously from uncommon serotypes. Gene names are given (where possible) for the schematic of each serotype, as well as the genetic locus tag of each strain being provided. Serotype names are indicated on the left, with strain names in the brackets. The O-antigen structures of O:1, O:2, O:3 [13], and O10 [18] are also shown. Regions conserved between strains are indicated as light pink blocks. ^a^The O:3 antigen gene cluster is drawn from nucleotide sequence under accession number Z118920; ^b^The genome of IP2222 (O:36) was annotated in this study; *Truncated gene.

Consequently, we selected one isolate within each serotype as a type strain for further analysis and found the genetic loci of these O-AGCs exhibited two patterns: within the *hemH-gsk* locus or somewhere else (Fig. 3). In addition, the O-AGCs shared by O:1 and O:2 were similar to that of O:3, and their O-AGCs were located between or adjacent to insertion sequence (IS), as was also the case in O:5 and O:5,27. Another common feature of the above-mentioned serotypes, along with O:9 and O:10 (strain WL-21 in this study), is that the O-AGCs were all located outside the *hemH-gsk* locus, and that their O-antigens seem to be synthesized by the ABC transporter dependent pathway. The putative O-AGCs of serotypes O:4, O:7/O:19, O:13, and O:21 were all mapped between *hemH* and *gsk*, as was also the case with O:8, and genes in each serotype were transcribed from *hemH* to *gsk*. However, in the case of O:36, the putative O-AGC genes were located between *hemH* and *gsk*, with the exception of four genes which were upstream of *hemH* and transcribed in the opposite direction. Another probably shared characteristic of these serotypes was that they produce their O-antigens via the Wzx/Wzy dependent pathway, except in the case of O:7/O:19. In particular, all strains of the latter pattern had a DDH sugar synthesis gene set which was present at the 5’ end within their O-AGCs (Fig. 3). All these features resembled those found in *Y. pseudotuberculosis* [55]. This evidence implied that the O-AGCs of *Y. enterocolitica* serotypes with *hemH-gsk* patterns, and those of *Y. pseudotuberculosis*, very likely originated from a common ancestor, followed by separate evolutionary events under the pressure of different niches, whereas the O-AGCs with the *wzm/wzt* gene set underwent a distinct evolutionary history.

Intriguingly, strains of O:7/O:19 possessed *wzm/wzt* and *wzx/wzy* simultaneously within their *hemH-gsk* loci (Fig. 3); this has not been previously reported among the O-AGCs of *Y. enterocolitica* and other *Yersinia* species. In a *Vibrio cholera* strain, the co-location of O-AGC and capsular synthesis genes containing both *wzm/wzt* and *wzx/wzy* was revealed, suggesting co-evolution of new O- and capsular-antigens [56]. However, no evidence of capsule has been reported in *Y. enterocolitica*. Therefore, the elucidation of the O-antigen processing mechanism in O:7/O:19 may arouse more interests in our next study. Another piece of evidence which attracted our attention was that none of the serotype O:6 isolates possessed standard O-AGC; only the outer core gene cluster within the *hemH-gsk* locus was discovered. In a few species, for example, in *Vibrio parahaemolyticus*, the O-antigen is deficient in the LPS molecule and the antigenic scheme is based on the variation of core oligosaccharide [57]. Thus, we propose that the antigenic property of O:6 may be determined by its core oligosaccharide, instead of O-antigen. This hypothesis in *Y. enterocolitica* O:6 could be addressed by fully elucidating the LPS structure.

### Strains of Serotypes are Grouped according to Their Virulence Profiles

**Fig. 4:**
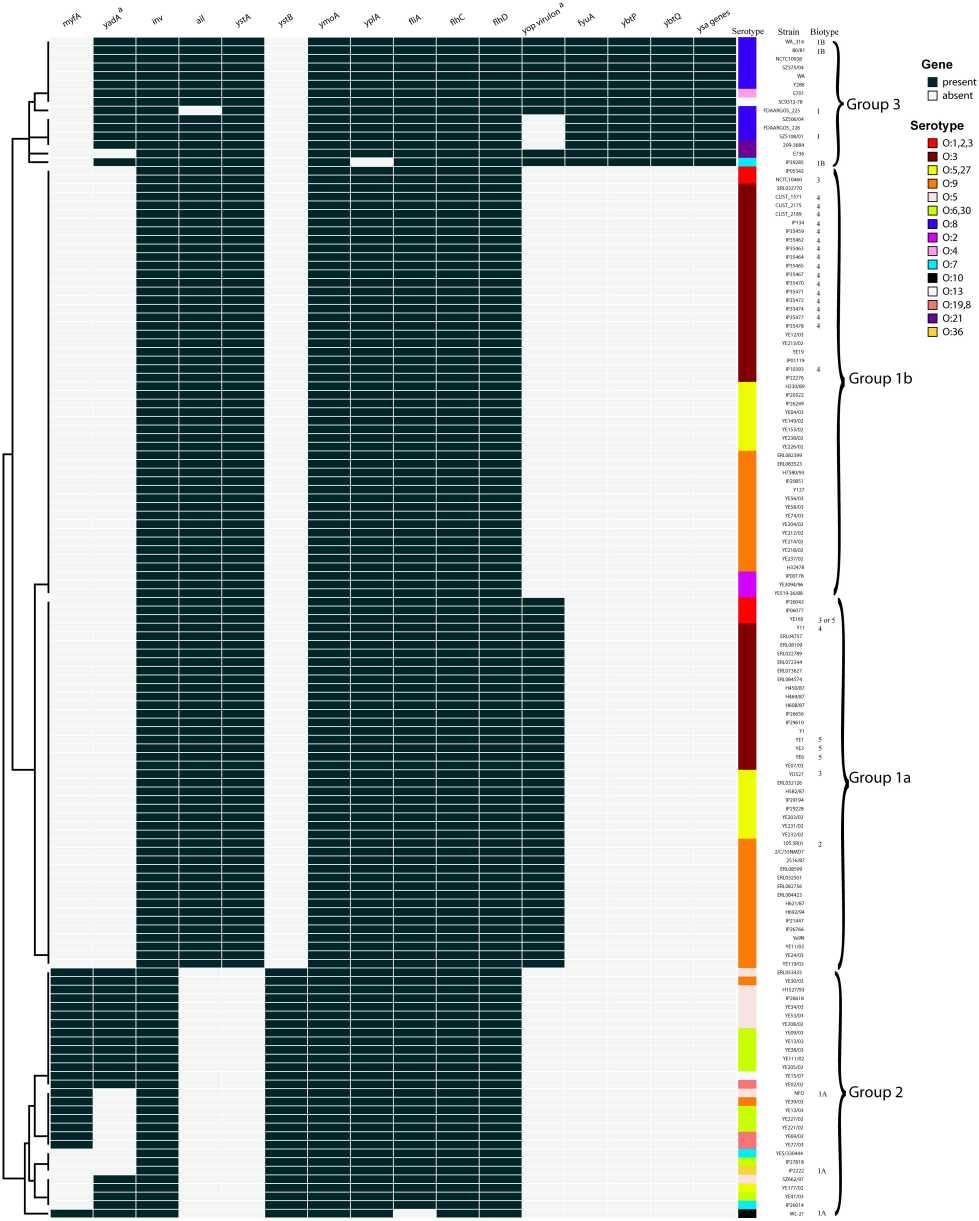
Virulence profiles of strains with serotypes derived from GenBank data. From left to right: virulence genes, with black indicating presence and white indicating absence; serotype highlighted in different colors; strain name with biotype from meta data if available. ^a^Plasmid-borne genes.

A total of 138 genomes representing 15 serotypes were investigated for virulence gene screening (Fig. 4). Among these virulence genes, *inv*, *ymoA*, *yplA*, *filA*, *flhC*, and *filD* were distributed in all or almost all isolates; no differences among serotypes were therefore exhibited. According to the pattern of other virulence genes, the 15 serotypes could be clearly divided into three groups: Group 1, consisting of serotypes O:1, O:2, O:3, O:5,27 and O:9; Group 2, mainly composed of serotypes O:5, O:6,30, and a few other minor serotypes including O:10 of this study; and Group 3, with serotype O:8 as its main member, along with O:4, O:21, one strain of O13, and one strain of O7 (Fig. 4).

In Group 1, the largest group, all isolates were characterized by the presence of *ail* and *ystA*, and the absence of *myfA*, *yadA*, *ystB*, *fyuA*, *ybtP*, *ybtQ*, and *yas* genes. Clearly, isolates of Group 1 could be further divided into two subgroups: 1a and 1b. Except for serotype O:2 strains which were located in Group 1b, other strains assigned to serotypes O:1, O:3, O:5,27 and O:9 were distributed into two subgroups. Obviously, the two subgroups were differentiated only by the presence/absence of the *yop* virulon. The *yop* virulon is the core of *Yersinia* pathogenicity machinery and is located within the Pyv [33]; unfortunately, it may be lost during prolonged storage, frequent passaging, or temperatures higher than 37°C [58], which, we propose, may lead to the absence of *yop* virulon in Group 1a. *myfA*, encoding a factor important for the beginning of infection, was originally found in bioserotype 4/O:3 strains isolated from clinical cases of yersiniosis, as well as some biotype 1A strains from patients with diarrhea [59]; however, no *myfA* was found in the O:3 strains in this study.

Within Group 2, all isolates possessed *ystB* instead of *ystA*, and most isolates possess *myfA* and *yadA*, the two genes absent in all isolates of Group 1. While *ystB*, instead of *ystA*, is known as a classical virulence marker of biotype 1A strain [19], the strong implication of *ystB* in serotypes O:5 and O:6, the main members of Group 2, has not been recorded previously. Another feature of Group 2 is that none of the isolates held *ail* or *yop* virulons. In contrast to the situation in subgroup 1a, the absence of *yop* virulon in Group 2 was unlikely to be attributed to the loss of PYv, since none of the Group 2 isolates were *yop* virulon positive. In terms of clinical manifestation, O:5/O:6 strains may partially or wholly lose the ability to cause the formation of necrotic abscesses; this is mainly mediated by the functionality of Pyv [60].

The main characteristics of Group 3 were that *fyuA*, *ybtP*, *ybtQ*, and yas genes were only present in the relevant isolates of this group, and thus the virulence factors were most abundant. *fyuA*, *ybtP* and *ybtQ* are the most representative genes of a pathogenicity island termed the high-pathogenicity island (HPI), and most of the genes of HPI are involved in the biosynthesis, transport, and regulation of yersiniabactin [61]. In addition, *yas* genes located within a Ysa pathogenicity island (Ysa-PI) encoding another T3SS play an important role in the colonization of gastrointestinal tissues during the earliest stage of infection [62]. HPI and Ysa-PI have been reported to be present only in *Y. enterocolitica* biotype 1B, the highly virulent biotype [63]. Our genome-wide analysis also showed that these two PIs were only present in isolates of Group 3 (three of these were assigned to biotype 1B), suggesting that strains of Group 3 must have unique pathogenic characteristics and mechanisms.

Herein, the *in silico* O-AGC characterization of non-prevalent serotypes provides the genetic basis for the design of novel geno-serotyping targets, and for the development of novel assays for subtyping and epidemiological surveillance. The revealing of different patterns of O-AGC locations indicates that there must be distinct evolutionary route among *Y. enterocolitica* strains, the mechanism of which needs further investigation. The O-antigen is a key virulence determinant, and its role in the pathogenesis of *Y. enterocolitica* has also been identified [26, 27]. In particular, the relationship between certain serotypes and specific virulence factors has been investigated in several bacteria species, for instance, in enterohemorrhagic *E. coli* O157:H7 [64]. Moreover, the regulation role of O-antigen in pathogenic promotion and environmental tolerance has also been confirmed [65, 66]. Here, we revealed a strong association between O-serotypes and the profile of virulence genes in *Y. enterocolitica* as a whole; this finding, we believe, will advance research into the role of O-antigens and other virulence factors, including their synergistic effect in the pathogenicity of this important food-borne pathogen.

## Figures and Tables

**Table 1:** Strains, plasmids, and primers used in this study.

Strain/plasmid/primer	Description
Strain	
WL-21	*Yersinia enterocolitica* 1A/O:10
WL-21Δ*wzm*	WL-21 *wzm* gene deletion
WL-21Δ*wzm*::*wzm*	WL-21Δ*wzm* complemented with pBAD33-wzm
S-17	*Escherichia coli* S17-1/λpir strain
Plasmid	
pRE112	Allelic exchange vector with *sacB*, chloramphenicol resistant
pRE112-updown	pRE112 fused with WL-21 *wzm* up- and down- homologous sequences
pBAD33	*araC*, promoter P_BAD_, chloramphenicol resistant
pBAD33-wzm	pBAD33 inserted with WL-21 *wzm* gene
Primer	Nucleotide sequences (5'-3')^a^
112F	TACACTCGTTAGCATTTACCAGCACCCTGATAAATGCTTCAATAATGG, forward primer for pRE112 linearization
112R	TACAGCGGCTCCTACTGAGGGTCAACAGCTCATTTCAGAATGG, reverse primer for pRE112 linearization
upF	CCATTATTGAAGCATTTATCAGGGTGCTGGTAAATGCTAACGAGTGTA, forward primer for *wzy* upload sequence
upR	CTGAACCCACATAGGTAGAATAGATACACGAGCCACAGAGGTCCAAG, reverse primer for *wzy* upload sequence
downF	CTTGGACCTCTGTGGCTCGTGTATCTATTCTACCTATGTGGGTTCAG, forward primer for *wzy* download sequence
downR	CCATTCTGAAATGAGCTGTTGACCCTCAGTAGGAGCCGCTGTA, reverse primer for *wzy* download sequence
wzm-cF	CGG*GGTACC***AGGAGG**AAAAGTGAAAGAGATGTTATTAGCGAT, forward primer for *wzm* cloning, digested with KnpI
wzm-cR	CCC*AAGCTT*TCATAATTCATCTACCATATCTTTG, reverse primer for *wzm* cloning, digested with HindIII

^a^Boldface characters indicate the Shine–Dalgarno box, and restriction sites are in italics.

**Table 2:** Details of proposed functions of the putative WL-21 O-AGC.

orf No.	Gene name	Species (Accession No.)	% Identical/% Similar	Putative function of protein
1	*orf1*	*Y. enterocolitica* (WP_005168322.1)	99/100	TerC family protein
2	*manC*	*Y. enterocolitica* (WP_076707092.1)	98/99	Mannose-1-phosphate guanylyltransferase/ mannose-6-phosphate isomerase
3	*manB*	*Y. enterocolitica* (WP_077174857.1)	99/100	Phosphomannomutase
4	*wzm*	*Y. enterocolitica* (WP_260505577.1)	90/96	ABC transporter permease
5	*wzt*	*Y. enterocolitica* (WP_221866130.1)	91/95	ABC transporter ATP-binding protein
6	*ubiG*	*Y. enterocolitica* (WP_221866131.1)	70/81	Class I SAM-dependent methyltransferase
7	*gtr1*	*Y. enterocolitica* (WP_221866132.1 )	79/89	Glycosyltransferase family 1 protein
8	*gmd*	*Y. enterocolitica* (WP_221866133.1 )	98/99	GDP-mannose 4,6-dehydratase
9	*rmd*	*Y. enterocolitica* (WP_076707088.1 )	93/96	GDP-mannose 4,6-dehydratase
10	*gtr2*	*Y. enterocolitica* (WP_076707087.1 )	88/94	Glycosyltransferase family 1 protein
11	*gtr3*	HDL6886110.1 )	93/97	Glycosyltransferase family 4 protein
